# Glucose-sensitive acetylation of Seryl tRNA synthetase regulates lipid synthesis in breast cancer

**DOI:** 10.1038/s41392-021-00714-0

**Published:** 2021-08-16

**Authors:** Jin Zhao, Hangming Bai, Xiaoyu Li, Jie Yan, Gengyi Zou, Longlong Wang, Xiru Li, Ze Liu, Rong Xiang, Xiang-Lei Yang, Yi Shi

**Affiliations:** 1 School of Medicine, Nankai University, Tianjin, China; 2 Department of General Surgery, Chinese PLA General Hospital, Beijing, China; 3 The Scripps Research Institute, La Jolla, CA USA

**Keywords:** Breast cancer, Cancer metabolism

## Abstract

Abnormally enhanced de novo lipid biosynthesis has been increasingly realized to play crucial roles in the initiation and progression of varieties of cancers including breast cancer. However, the mechanisms underlying the dysregulation of lipid biosynthesis in breast cancer remain largely unknown. Here, we reported that seryl tRNA synthetase (SerRS), a key enzyme for protein biosynthesis, could translocate into the nucleus in a glucose-dependent manner to suppress key genes involved in the de novo lipid biosynthesis. In normal mammary gland epithelial cells glucose can promote the nuclear translocation of SerRS by increasing the acetylation of SerRS at lysine 323. In SerRS knock-in mice bearing acetylation-defective lysine to arginine mutation, we observed increased body weight and adipose tissue mass. In breast cancer cells the acetylation and nuclear translocation of SerRS are greatly inhibited. Overexpression of SerRS, in particularly the acetylation-mimetic lysine to glutamine mutant, dramatically inhibits the de novo lipid synthesis and hence greatly suppresses the proliferation of breast cancer cells and the growth of breast cancer xenografts in mice. We further identified that HDAC4 and HDAC5 regulated the acetylation and nuclear translocation of SerRS. Thus, we identified a SerRS-meditated inhibitory pathway in glucose-induced lipid biosynthesis, which is dysregulated in breast cancer.

## Introduction

Highly activated lipid biosynthesis, in particularly the de novo fatty acid (FA) biosynthesis, has been demonstrated in many types of tumors even in the presence of exogenous lipid sources, which plays essential roles in tumor development, progression and drug resistance by generating a diverse intracellular lipid pool for the synthesis of biological membranes, energy storage and the production of important signaling molecules.^1–6^ Increased uptake of glucose and glutamine in cancer cells contributes the carbon source for the production of citrate, which is converted to acetyl-CoA by ATP-citrate lyase (ACLY) and subsequently carboxylated to malonyl-CoA by acetyl-CoA carboxylases (ACACs, also known as ACCs), the rate-limiting step committing acetyl-CoA to FA biosynthesis.^7–9^ Then seven malonyl-CoA molecules are condensed by fatty acid synthase (FASN) to generate palmitate (FA 16:0), which can be further elongated or desaturated to form other FA species. Aectyl-CoA is also the building block for the synthesis of cholesterol and steroid hormones, where it is converted to acetoacetyl-CoA pool by acetoacetyl-CoA thiolase 2 (ACAT2) for the generation of cholesterol and steroids.^10^ In this pathway, mevalonate production is the committed step catalyzed by 3-hydroxy-3-methylglutaryl-CoA synthase 1 (HMGCS1) and 3-hydroxy-3-methylglutaryl-CoA reductase (HMGCR), which is also the key regulated step in the de novo synthesis of cholesterol. Metabolically stressed cancer cells can also utilize exogenous acetate to generate acetyl-CoA by acyl-CoA synthetase short chain family member 1 and 2 (ACSS1 and ACSS2).^11,12^ Upregulation of key genes involved in de novo FA synthesis and lipid metabolism has been well demonstrated to be a metabolic feature of cancer cells.^2^

In case of breast cancer, the lipid metabolic feature differs among different subtypes of breast cancers classified according to the expression of hormone receptors or growth factor receptors. The receptor positive breast cancers show elevated genes involved in de novo lipid synthesis, FA metabolism and oxidation, while triple-negative breast cancers (TNBCs) have increased expression of genes in exogenous lipid uptake and storage as well.^13^ In human epidermal growth factor receptor 2-enriched (HER2 + ) breast cancers, highly activated HER2-PI3K-AKT-mTORC1 signaling results in the activation of the transcriptional factor sterol regulatory element-binding factor 1 (SREBF1, also known as SREBP1) to activate the expression of key lipogenic genes, including *FASN*, *ACLY*, and *ACACA* (also known as *ACC1*), and consequently upregulates de novo lipid synthesis that contributes to the aggressiveness of HER2 + breast cancers.^14–16^ Oncogenic signaling activated lipogenic enzymes, such as ACLY and ACSS2, can in turn affect the cancer epigenome by generating acetyl-CoA to change the global histone acetylation level.^17,18^ Nutrient deficiency and hypoxia can induce the formation of lipid droplets for the storage of fatty acids and cholesterol in the form of triglycerides and cholesterol esters, which can be mobilized to fuel the survival, proliferation and migration of TNBC under metabolic stress.^13,19,20^ Although, some abnormal lipid metabolism phenotypes in breast cancers have been attributed to the dysregulation on the major pathways regulating metabolism, such as the hyperactive PI3K-AKT signaling, the regulatory mechanisms underlying the very complicated lipid metabolic rewiring that favors the malignancy of different subtypes of breast cancer remains largely unknown.

In this study, we reported a novel function of SerRS in regulating lipid biosynthesis. SerRS belongs to the aminoacyl tRNA synthetases (AARSs) family with the classical function of ligating serine to its cognate tRNA for protein biosynthesis. During evolution, SerRS in vertebrates gained a unique carboxyl-terminal domain (UNE-S domain) harboring a nuclear localization signal, allowing the nuclear presence of 10% of SerRS protein (~200 nM, near the Kd value of SerRS and DNA interaction),^21^ where it functions as a transcriptional repressor to compete with c-Myc for the regulation of vascular endothelial growth factor A (VEGFA) transcription and the vascular homeostasis.^22^ In addition to bind on *VEGFA* promoter, we also found that SerRS was able to directly bind on telomere DNA repeats and interacts with POT1 to regulate telomere length and cellular senescence, suggesting a broad function of SerRS in the nucleus.^23^ Here, we systematically investigated the biological significance of nuclear SerRS, especially in breast cancer due to that high expression of SerRS correlates with better clinical outcome of breast cancer patients,^23^ suggesting a novel function of SerRS beyond protein synthesis in suppressing tumor progression. We found that the nuclear translocation of SerRS was regulated by the glucose level-affected acetylation of SerRS at lysine 323 residue (K323). By using chromatin immunoprecipitation followed by DNA deep-sequencing (ChIP-Seq) and transcriptome analysis, we identified many SerRS-regulated genes involved in the de novo lipid biosynthesis. And SerRS knock-in mice bearing acetylation-defective lysine to arginine (K323R) mutation in one allele of SerRS gene showed decreased nuclear SerRS localization, higher body weight and elevated adipose tissue mass, supporting that nuclear SerRS regulates lipid metabolism. Furthermore, we found that in TNBC cells, the acetylation and the nuclear translocation of SerRS were not affected by glucose and forced overexpression of SerRS in the nucleus dramatically decreased the lipid synthesis and ultimately suppressed the proliferation of TNBC cells and the growth of TNBC xenografts in mice. Thus, we discovered a SerRS-mediated pathway regulating glucose-fueled lipid biosynthesis and its important role in the rewiring of lipid metabolism in TNBC.

## Results

### Glucose regulates the nuclear translocation of SerRS by acetylation which is dysregulated in breast cancer cells

Although the nuclear translocation of SerRS and its significance in vascular development have been demonstrated in zebrafish,^21,22^ our recent study showed that SerRS was able to promote telomere shortening and trigger the senescence of tumor cells,^23^ suggesting an important role of nuclear SerRS against tumor development. However, it keeps unknown if and how the nuclear import of SerRS is regulated. We therefore first examined the nuclear localization of SerRS under different metabolic stresses that cancer cells encounter during their growth and progression, including hypoxia and deficiency of major nutrients.

**Fig. 1:**
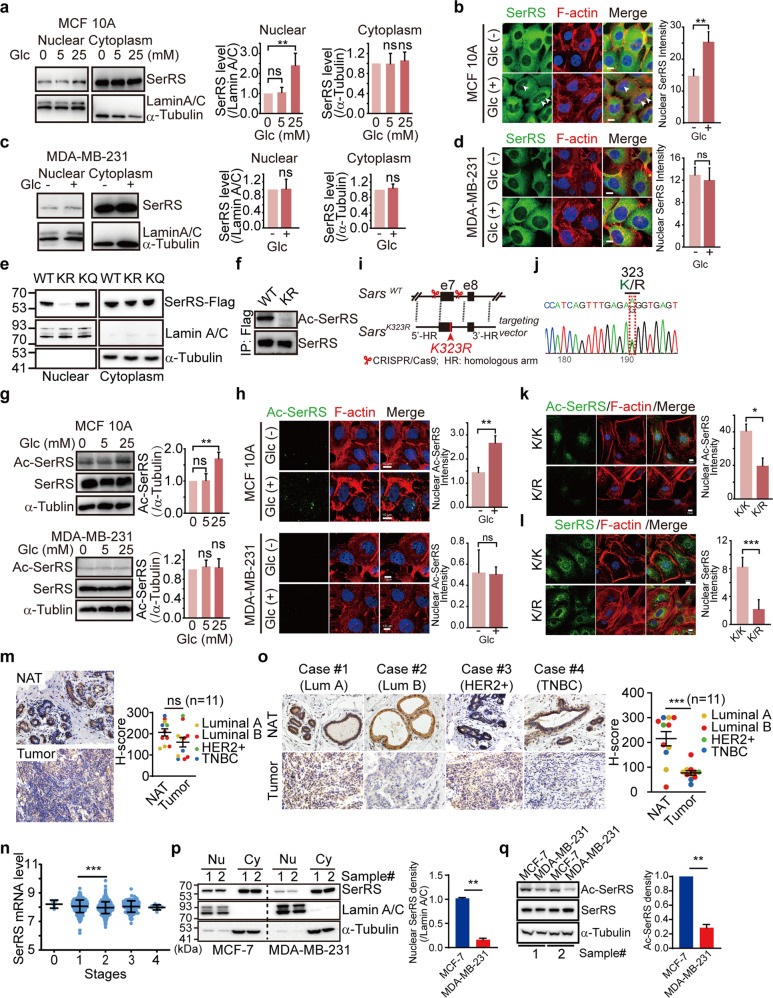
Glucose-induced acetylation of SerRS affects its nuclear translocation. **a** MCF 10A cells were cultured in the medium containing indicated dosages of glucose (Glc) for 24 h and the SerRS in the cytoplasm and nuclear fractions were analyzed by western blot (left panel). Nuclear Lamin A/C and cytoplasm α-tubulin were used to show the purities of nuclear and cytoplasm fractions. Quantification results (right panels) are shown as means ± SEM from three independent experiments (**P < 0.01, ns indicates not significant, by unpaired Student’s t-test). **b** Immunofluorescent staining to show the subcellular localization of SerRS in MCF 10A cells cultured in absence or presence of 25 mM Glc (scale bars represent 10 μm) and the quantification of nuclear SerRS (means ± SEM from three independent experiments, **P < 0.01, by unpaired Student’s t-test). **c**, **d** The nuclear translocation of SerRS in MDA-MB-231 cells cultured in the absence or presence of 25 mM Glc was analyzed by western blot (**c**) and immunofluorescent staining (**d**, Scale bars represent 10 μm). The quantification results are shown as means ± SEM from three independent experiments (ns indicates not significant, by unpaired Student’s t-test). **e** Western blot analysis to show the nuclear translocation of wild type (WT) SerRS and its mutants with Lysine 323 (K323) to arginine (KR) or to glutamine (KQ) mutations. **f** The specificity of customized antibody against acetylated SerRS at K323 (Ac-SerRS) was tested by western blot analysis on immunoprecipitated Flag-tagged WT and KR SerRS from MCF 10A cells. **g**, **h** The SerRS acetylation at K323 was analyzed by western blot (**g**) or immunofluorescent staining (**h**) using Ac-SerRS specific antibody in indicated cells cultured in different dosages of glucose. The quantification results are shown as means ± SEM from three independent experiments (**P < 0.01, ns indicates not significant, by two-sides Student’s t-test). In (h), the glucose dosages were 25 mM. Scale bars represent 10 μm. **i** Knock-in mice bearing Lys^323^-to-Arg heterozygote mutation (Sars^K/R^) was generated by CRISPR/Cas9 and homologous recombination of a targeting vector with mutation of K^323^-encoding sequence. **j** Validation of Sars knock-in mice with heterozygote Lys^323^-to-Arg mutation by PCR and sequence analysis. **k**, **l** Immunostaining of Ac-SerRS (**k**) and total SerRS (**l**) in fibroblast cells isolated from Sars^K/R^ mice. Scale bars represent 10 μm. The quantification results are shown as means ± SEM from three independent experiments (*P < 0.05, ***P < 0.001, by two-sides Student’s t-test). **m** Immunohistochemistry staining of SerRS in human breast cancer tissues and normal adjacent tissues (NAT) (left panels) and the quantification (right panel, means ± SEM, ns indicates not significant, by paired Student’s t-test). **n** Comparison of the SerRS mRNA levels in different stages of breast cancers (means ± SD, ***P < 0.0001, by unpaired Student’s *t*-test). Data were collected from TCGA data base. **o** Immunohistochemistry staining of Ac-SerRS in human breast cancer tissues and normal adjacent tissues (NAT) and the quantification (means ± SEM, ***P < 0.0001, by paired Student’s t-test). **p** Western blot analysis of nuclear (Nu) and cytoplasmic (Cy) SerRS in indicated breast cancer cell lines and the quantification (means ± SEM from two independent experiments, **P < 0.001, by unpaired Student’s t-test). **q** Western blot analysis of lysine 323-acetyated SerRS in indicated breast cancer cell lines and the quantification (means ± SEM from two independent experiments, **P < 0.001, by unpaired Student’s t-test)

We tested human normal mammary gland epithelial cell line MCF 10A for the subcellular distribution of SerRS in hypoxia and deficiency of major nutrients including glucose, glutamine, serine and acetate. We found that the nuclear localization of SerRS was not affected by glutamine, serine and acetate (Supplementary fig. S1a-c). Hypoxia does not affect the nuclear import of SerRS (data not shown). Interestingly, glucose can significantly increase the nuclear localization of SerRS in a dose-dependent manner in MCF 10A (Fig. 1a, b). In contrast, in breast cancer cells MDA-MB-231, SerRS is mainly localized in the cytoplasm and its nuclear import cannot be promoted by glucose, glutamine, serine and acetate at all (Fig. 1c, d, and Supplementary fig. S1d-f). These results suggest that in normal mammary gland epithelial cells, the nuclear localization of SerRS is regulated by the extracellular glucose level, which is somehow dysregulated in breast cancer cells.

We next investigated how the nuclear import of SerRS was regulated. Since protein acetylation is always affected by the level of acetyl-CoA, a key cellular metabolite connecting catabolism and anabolism,^12,18^ we then focused on the possible acetylation of SerRS that might affect SerRS nuclear localization. In a systemic identification of lysine acetylation by high-resolution mass spectrometry study, SerRS was shown to be acetylated at the lysine 323residue (K323) with unknown biological significance.^24^ We mutated K323 to arginine (K323R) and glutamine (K323Q) to mimic the unacetylated and acetylated SerRS, respectively. Our cell fractionation analysis showed that K323Q mutant could enter the nucleus as wild type (WT) SerRS did, while the K323R could no longer enter the nucleus (Fig. 1e), suggesting that only K323-acetylated SerRS was able to enter the nucleus. To further confirm, we made specific antibody against K323-acetylated SerRS whose specificity was confirmed by western blot analysis of unacetylated SerRS K232R mutant (Fig. 1f). By using this antibody, we analyzed the acetylation of endogenous SerRS and found that glucose did increase the acetylation of SerRS in MCF 10A cells in a dose-dependent manner, whereas in MDA-MB-231 cells SerRS remained to be unacetylated even in the presence of glucose (Fig. 1g). Further immunofluorescent staining showed that in MCF 10A glucose increased the acetylated SerRS that mainly localized in the nucleus, which was not observed in MDA-MB-231 cells (Fig. 1h).

To further get insights into the biological significance of SerRS acetylation at lysine 323 in vivo, we made SerRS acetylation-defective mice by mutating the lysine 323 residue of SerRS to arginine in C57BL/6 mice using CRISPR/Cas9 technology (Fig. 1i). Surprisingly, we only got heterozygote K323R mutant mice (Sars^K/R^) and homozygote mutation of K323R caused embryonic lethality (Fig. 1j), suggesting an essential role of SerRS acetylation at lysine 323 in the early embryo development. In the skin fibroblast cells of Sars^K/R^ mice, we confirmed K323R mutation caused dramatically decreased acetylation of SerRS compared with that in the cells from the wild type littermates (Sars^K/K^) (Fig. 1k). Consistently, the total nuclear SerRS protein level was dramatically decreased in Sars^K/R^ mice (Fig. 1l). These data strongly suggest that the nuclear localization of SerRS is regulated by acetylation at lysine 323 residue.

Unlike most other AARSs, SerRS mRNA level has been shown to positively correlate with the survival of breast cancer patients, suggesting a tumor suppressor role of SerRS beyond protein translation in the cytoplasm.^23^ We therefore analyzed the SerRS protein level in eleven human breast cancer tissues, which includes the intrinsic subtypes of luminal A and B, HER2 + and TNBC. We found almost no change in the total SerRS protein level in most subtypes of breast cancer patients when compared to the normal adjacent tissues except in TNBC, which showed decreased SerRS level (Fig. 1m). And in a cohort containing 1,403 breast cancer patients from the cancer genome atlas (TCGA), SerRS expression only showed a slight change during the cancer progression (Fig. 1n). We then examined the acetylation of SerRS in these eleven human breast cancer tissues since acetylation affects SerRS nuclear translocation which may impact the relevant nuclear function of SerRS. We found that in almost all subtypes of human breast cancer tissues the SerRS acetylation was dramatically decreased when compared with the normal adjacent tissues (Fig. 1o). In addition, both the nuclear SerRS level and lysine 323-acetylated SerRS are much lower in breast cancer cell line MDA-MB-231 (TNBC) than that in MCF-7 (luminal A) (Fig. 1p and q), suggesting an important role of dysregulated SerRS acetylation in the development of different subtypes of breast cancer.

### Defective SerRS acetylation at lysine 323 enhances adipose deposition in mice

**Fig. 2:**
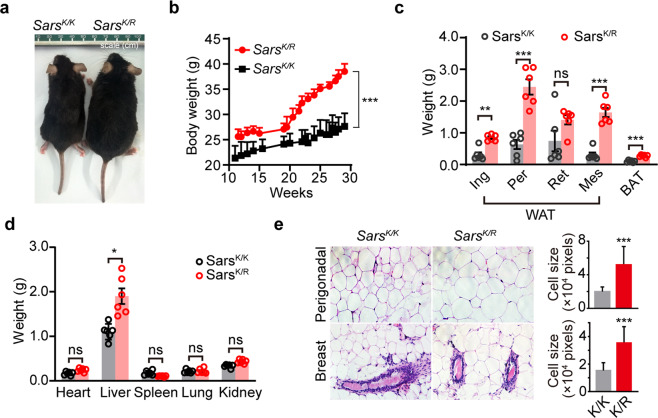
Acetylation-defective SerRS Lys^323^-to-Arg (Sars^K/R^) heterozygote knock-in mice exhibit increased body weight and adiposity. **a** Representative images of a Sars^K/R^ mouse showing bigger body size than the wild type Sars^K/K^ littermate at 25 weeks. **b** Growth curves of Sars^K/R^ and Sars^K/K^ mice (*n* = 8, mean ± SEM, ***P < 0.0001, by two-way ANOVA). **c** Weights of the inguinal (Ing), perigonadal (Per), retroperitoneal (Ret), and mesenteric (Mes) white adipose tissues (WAT) and interscapular brown adipose tissue (BAT) from 18-week female mice fed a standard diet (means ± SEM, *n* = 6, **P < 0.01, ***P < 0.001, ns indicates not significant, by unpaired Student’s t-test). **d** Weights of the indicated major organs from 18-week female mice fed a standard diet (means ± SEM, *n* = 6, *P < 0.05, ns indicates not significant, by unpaired Student’s t-test,). **e** Histological staining analysis of indicated WAT in 18-week old female Sars^K/R^ and Sars^K/K^ mice and the quantification (***P < 0.0001, by unpaired Student’s t-test)

We next investigated the biological significance of SerRS acetylation at lysine 323 in mice. We monitored the growth of Sars^K/K^ and Sars^K/R^ mice maintained on standard diet for near 30 weeks starting from 11 week of age. The Sars^K/R^ mice exhibit about 12-28% higher body mass than wild type Sars^K/K^ mice (Fig. 2a and b). The body length of Sars^K/R^ was the same as wild type. However, we observed significantly increased adipose tissue deposition in opened abdominal cavity of Sars^K/R^. The weight of major white adipose tissue (WAT) depots in Sars^K/R^ mice was about over two folds higher than that in Sars^K/K^ mice (Fig. 2c), while the weights of other major organs showed almost no change except that the weight of the liver increased a little bit (Fig. 2d), suggesting increased WAT as a major contributor to the higher body mass of Sars^K/R^ mice. The weight of interscapular brown adipose tissue (BAT) was also increased (Fig. 2c). Further hematoxylin and eosin staining on the section of WAT tissues showed dramatic increase in adipocyte size in Sars^K/R^ mice (Fig. 2e). These phenotypes of Sars^K/R^ mice suggested a role of SerRS acetylation on regulating lipid metabolism.

### Acetylation of SerRS greatly inhibits the progression of breast cancer

**Fig. 3:**
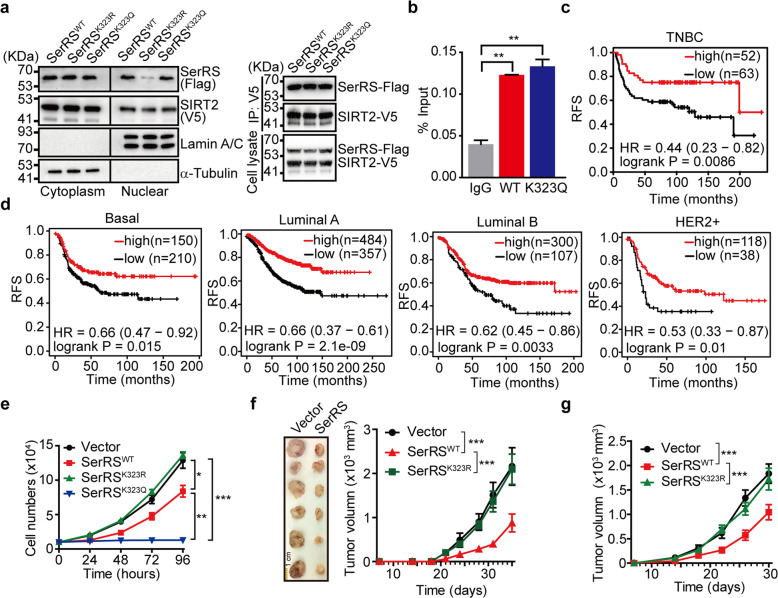
Acetylation of SerRS at Lys^323^ is essential for its tumor suppressor activity. **a** Western blot analysis of subcellular localization of SerRS and SIRT2 in MDA-MB-231 cells transfected with V5-tagged SIRT2 and Flag-tagged wild type (WT) or mutant SerRS, i.e., SerRS^K323R^ or SerRS^K323Q^ (left panel) and the coimmunoprecipitation assay using the whole cell lysates (right panel). **b** Chromatin immunoprecipitation assay in the lysates of MDA-MB-231 cells transfected with wild type SerRS (WT) or SerRS with K232Q mutation (means ± SEM from three independent experiments, **P < 0.01, by unpaired Student’s t-test). **c** Kaplan–Meier survival curve of 115 breast cancer patients with high and low SerRS protein levels. **d** Kaplan–Meier survival curves of indicated intrinsic subtypes of breast cancer patients with high and low SerRS mRNA levels. **e** The proliferation curves of MDA-MB-231 cells transfected with empty vector (Vector), WT SerRS, or mutant SerRS, i.e., SerRS^K323R^ and SerRS^K323Q^. Data are plotted as means ± SEM from three independent experiments (*P < 0.05, **P < 0.01, ***P < 0.001, by two-way ANOVA). **f** The growth curves of tumor xenografts formed by MDA-MB-231 cells stably transfected with empty vector (Vector), wild type SerRS (SerRS^WT^), or SerRS^K323R^ mutant (means ± SEM, *n* = 6, ***P < 0.001, by two-way ANOVA). **g** The growth curves of tumor homografts formed by 4T1 cells stably transfected with empty vector (Vector) or SerRS (means ± SEM, *n* = 7, ***P < 0.001, by two-way ANOVA,)

Given that acetylation of SerRS regulates its translocation to the nucleus, where SerRS can function as a transcriptional regulator,^22^ we postulated that SerRS might regulate key genes involved in lipid metabolism. Firstly, we checked if SerRS acetylation affected its nuclear interaction with its cofactor SIRT2 and DNA. Although the translocation of SerRS into the nucleus is affected by acetylation at lysine 323 (Fig. 3a, left panel), our coimmunoprecipitation using the whole cell lysates and ChIP assays using the nuclear lysates show that SerRS acetylation at lysine 323 does not affect its capacity to interact with SIRT2 or chromosome DNA (Fig. 3a, b, respectively), indicating that acetylation at lysine 323 doesn’t affect the activity of SerRS as a transcriptional repressor.

Since lipid de novo synthesis have been shown to be highly activated in cancer cells, especially in breast cancer cells, to facilitate rapid cell proliferation, the defective SerRS acetylation causes its less nuclear import (Fig. 1k, l) and consequently increased adiposity (Fig. 2c, e) suggest that defective acetylation and nuclear translocation of SerRS in breast cancer cells (Fig. 1c, d and o) may play important roles in abnormal activation of lipid synthesis.

Higher mRNA level of SerRS has been shown to correlate with better clinical outcome of breast cancer patients due to its nuclear functions.^23^ We also analyzed the SerRS protein levels in a recently reported cohort containing 115 TNBC subtype of breast cancer patients,^25^ and found that higher SerRS protein level tightly correlated with better relapse-free survival (RFS) (Fig. 3c). Further analysis based on TCGA database shows that higher SerRS mRNA level correlates with better RFS of other subtypes of breast cancer including basal, luminal A, luminal B and HER2 + (Fig. 3d). These results strongly indicate that the nuclear function of SerRS is important to suppress the development of different subtypes of breast cancer.

To further confirm the role of SerRS in breast cancer development, we overexpressed SerRS in mouse breast cancer cells 4T1 and human breast cancer cells MDA-MB-231, which was able to force more SerRS proteins to enter the nucleus. Overexpression of SerRS dramatically inhibited the proliferation of MDA-MB-231 cells, while SerRS K323R mutant showed no effects on cell proliferation (Fig. 3e). Strikingly, overexpression of SerRS K323Q mutant totally blocked cell proliferation (Fig. 3e), suggesting a potent inhibitory activity of nuclear SerRS on cell proliferation. We further inoculated these cells subcutaneously into mice. As expected, SerRS overexpression significantly inhibited the growth of both MDA-MB-231 xenografts and 4T1 homografts in mice (Fig. 3f, g). However, overexpression of SerRS K323R mutant showed no effect on the growth of tumor xenografts (Fig. 3f, g). Breast cancer cells overexpressing SerRS K323Q mutant stopped proliferation and failed to form tumor xenograft in mice. These results suggested a robust role of nuclear SerRS in suppressing breast cancer development.

### SerRS transcriptionally regulates key genes involved in lipid metabolism

**Fig. 4:**
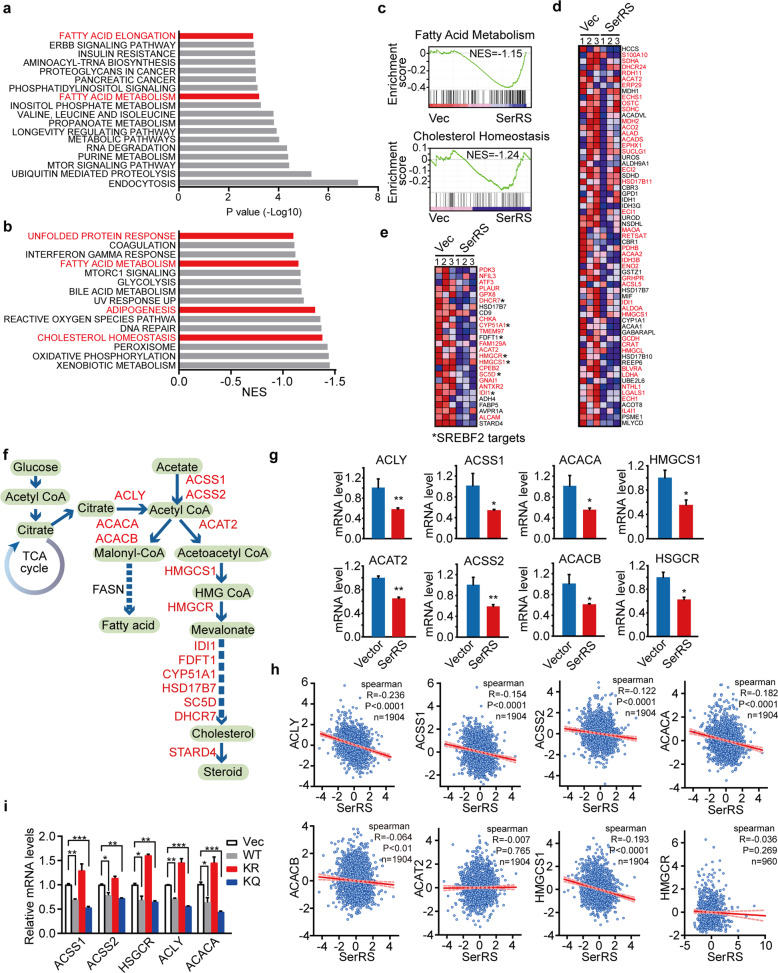
SerRS transcriptional suppresses key genes involved in the de novo lipid biosynthesis. **a** The top KEGG pathways that SerRS target genes identified by chromatin immunoprecipitation and DNA deep sequencing (ChIP-Seq) enrich in. **b** The top enriched pathways by GSEA analysis of the SerRS-suppressed genes in MDA-MB-231 cells identified by RNA sequencing. **c** GSEA plot of the fatty acid metabolism and cholesterol homeostasis pathways based on transcriptomes of empty vector-transfected versus SerRS-overexpressed MDA-MB-231 cells. NES, normalized enrichment score. **d**, **e** Heatmaps of the mRNA levels of enriched genes in fatty acid metabolism pathway (**d**) and cholesterol homeostasis pathway (**e**). The red signal denotes high level and the blue signal denotes low level in SerRS-overexpressed MDA-MB-231 cells. The genes in red were also SerRS target genes identified by ChIP-Seq. **f** The de novo lipid synthesis pathways and the key enzymes. **g** The mRNA levels of indicated genes in SerRS-overexpressed and empty vector-transfected MDA-MB-231 cells were further quantified by qRT-PCR (means ± SEM, *n* = 3, Student’s *t*-test, *P < 0.05, **P < 0.01). **h** The correlation of mRNA levels of SerRS and the indicated key genes in human breast cancers collected from TCGA database. **i** The mRNA levels of indicated genes in MDA-MB-231 cells transfected with empty vector (Vec), wild type or mutant SerRS were quantified by qRT-PCR (means ± SEM, *n* = 3, Student’s *t*-test, *P < 0.05, **P < 0.01, ***P < 0.001)

To get insights into the molecular mechanism underlying the tumor suppressor function of SerRS, we overexpressed Flag-tagged SerRS in MDA-MB-231 cells, which were analyzed by ChIP-Seq to identify candidate genes directly regulated by SerRS. Among all the SerRS binding sites on the genomic DNA, around 4.5% sites are within the −2 kb to +2 kb region around the transcriptional start sites, 48.6% sites within the introns and 0.6% sites within the exons and the rest 46.3% sites are in the intergenic regions. We found 11,420 protein-encoding genes which contained SerRS binding sites on their promoter and/or coding regions (Supplementary Table S1). Pathway enrichment analysis of these candidate target genes of SerRS showed that SerRS might regulate fatty acid metabolism and the major metabolism-regulating mTOR signaling pathway which support the abnormal adiposity phenotype of Sars^K/R^ mice (Fig. 4a).

To further confirm, we also performed transcriptome analysis on SerRS-overexpressed MDA-MB-231 cells and empty vector transfected control cells (Supplementary Table S2). Gene set enrichment analysis (GSEA) of the transcriptomes also showed hallmark gene sets involved in adipogenesis and lipid metabolism, including fatty acid metabolism and cholesterol metabolism, and unfolded protein response that regulates fatty acid metabolism^26,27^ were inhibited by SerRS (Fig. 4c–e). Most of these genes also contained SerRS binding sites (Fig. 4d and e, Supplementary fig. S2). We next chose genes encoding the key enzymes involved in the de novo synthesis of fatty acid and cholesterol to further confirm by quantitative RT-PCR (qRT-PCR), which include ACLY, ACACA/ACACB, ACSS1/ACSS2, ACAT2, HMGCS1 and HMGCR (Fig. 4f). Our ChIP-Seq results showed that all these key genes contained the SerRS binding sites (Supplementary fig. S2). Our qRT-PCR results confirmed that overexpression of SerRS in MDA-MB-231 cells suppressed the expression of these genes (Fig. 4g). And we also analyzed the transcriptomes of breast cancer patients in TCGA database and found that the expression of SerRS negatively correlated with most of these key genes involved in lipid synthesis (Fig. 4h), supporting that SerRS was a transcriptional repressor of these genes.

To investigate if the inhibitory role of SerRS on the expression of lipid synthesis genes is due to its nuclear function, we tested the SerRS K323R mutant that was not able to enter the nucleus (Fig. 1e). Compared with wild type SerRS and SerRS K323Q mutant, SerRS K323R mutant could no longer inhibit the expression of key genes in lipid synthesis (Fig. 4i), suggesting that SerRS acetylation on lysine 323 was a new mechanism for the regulation of lipid synthesis.

### SerRS inhibits the de novo lipid synthesis in breast cancer cells

**Fig. 5:**
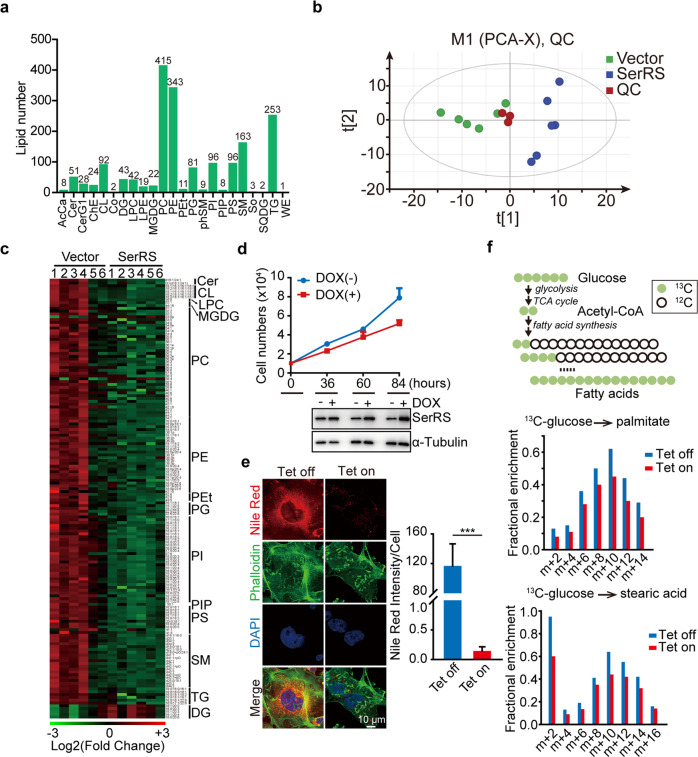
SerRS inhibits the de novo lipid synthesis in breast cancer cells. **a** The lipid species identified in the metabolomics analysis of MDA-MB-231 cells stably transfected with empty vector or SerRS. AcCa acyl carnitines; Cer ceramides; CerG1 glucosylceramides; ChE cholesteryl esters; CL cardiolipins; Co coenzyme; DG diradylglycerolipids; LPC lysophosphatidylcholines; LPE lysophosphatidylethanolamines; MGDG monogalactosyldiacylglycerols; PC phosphatidylcholines; PE phosphatidylethanolamines; PEt phosphatidylethanols; PG phosphatidylglycerols; phSM phytosphingosines; PI phosphatidylinositols; PIP phosphatidylinositol phosphates; PS phosphatidylserines; SM sphingomyelins; So sphingosines; SQDG sulfoquinovosyldiacylglycerols; TG triradylglycerolipids; WE wax esters. **b** Principal component analysis (PCA) of lipids identified in MDA-MB-231 cells transfected with empty vector or SerRS (*n* = 6). QC quality control. **c** Heatmap of lipid species with significant changes (*p* < 0.05, *n* = 6) in SerRS- overexpressed versus control MDA-MB-231 cells. **d** Cell proliferation curves of MDA-MB-231 cells transfected with Tet-inducible SerRS which was induced by 1 mg/ml of doxycycline (DOX). The SerRS expression was monitored by western blot (low panel). **e** The lipid droplets (LDs) in MDA-MB-231 cells with Tet-induced SerRS were shown by immunofluorescent staining using Nile red (red). Actin filaments were counterstained with phalloidin (green). The LDs were quantified by measuring the intensity of red fluorescent signals (means ± SEM, *n* = 6, Student’s t-test, ***P < 0.0001). **f** Metabolic flux analysis of SerRS-inducible MDA-MB-231 cells labeled by ^13^C-glucose that is utilized for the de novo fatty acid synthesis (upper panel). The incorporation of ^13^C into palmitate and stearic acid in SerRS-induced (Tet on) and uninduced MDA-MB-231 cells were shown in the low panels

To confirm the role of SerRS in regulating lipid synthesis in breast cancer cells, we performed steady-state lipid metabolomics (lipidomics) analysis on SerRS-overexpressed MDA-MB-231 cells. We identified 1,812 lipids belonging to 23 lipid classes (Fig. 5a, Supplementary Table S3). Principal component analysis (PCA) results showed that the lipidomics of SerRS-overexpressed MDA-MB-231 was quite different compared with empty vector transfected cells (Fig. 5b). We observed a general decrease of most lipids identified upon SerRS overexpression (Supplementary fig. S3). The significantly changed lipid species (≥2 folds) in SerRS overexpressed MDA-MB-231 cells were shown in Fig. 5c.

To further confirm the inhibitory role of SerRS in the de novo lipid synthesis from glucose-sourced carbon, we analyzed the metabolic flux by feeding the MDA-MB-231 cells with ^13^C-labeled glucose. We first established a SerRS-inducible MDA-MB-231 cells and confirmed that induced overexpression of SerRS significantly inhibited the cell proliferation (Fig. 5d). Also, once SerRS overexpression was induced, we observed dramatically reduced intracellular lipid droplets in MDA-MB-231 cells shown by Nile red staining (Fig. 5e), further confirming the inhibitory role of SerRS in lipid synthesis. We next traced the metabolic fluxes in SerRS-induced MDA-MB-231 cells and observed generally decreased intermediates in fatty acid de novo synthesis pathways (Fig. 5f and Supplementary Table S4), further confirming that SerRS inhibits the de novo synthesis of lipids.

### HDAC4/5 regulates SerRS acetylation and nuclear localization

**Fig. 6:**
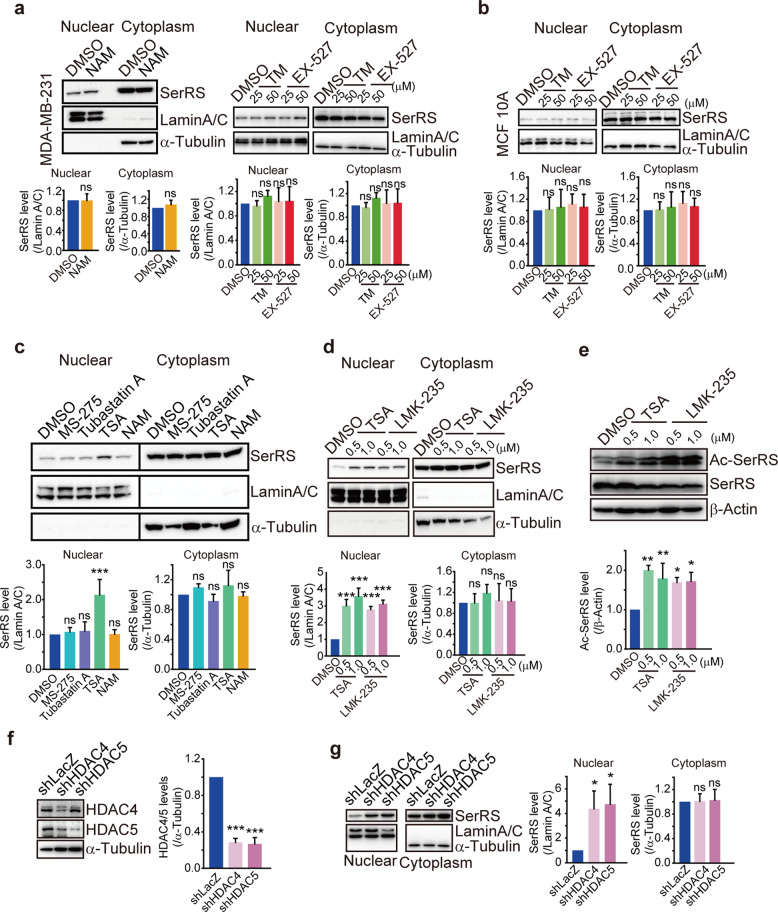
HDAC4 and HDAC5 regulate the acetylation of SerRS in breast cancer cells. **a**, **b** The MDA-MB-231 cells (**a**) and MCF 10A cells (**b**) were treated with SIRT inhibitor nicotinamide (NAM, 10 mM), SIRT1 inhibitor selisistat (EX-527), and SIRT2 inhibitor thiomyristoyl (TM) at indicated dosages for 48 h and the nuclear and cytoplasmic SerRS were analyzed by western blot. Quantification results are shown as means ± SEM from three independent experiments (ns indicates not significant, by unpaired Student’s t-test). **c** The MDA-MB-231 cells were treated with HDAC inhibitor trichostatin A (TSA, 1 μM), HDAC1 and HDAC3 inhibitor entinostat (MS-275, 10 μM), HDAC6 inhibitor Tubastatin A (10 μM), NAM (10 mM) and DMSO control for 48 h and the nuclear and cytoplasmic SerRS were analyzed by western blot. Quantification results are shown as means ± SEM from three independent experiments (***P < 0.001, by unpaired Student’s t-test). **d** Western blot analysis of nuclear translocation of SerRS in MDA-MB-231 cells treated with TSA and specific HDAC4 and HDAC5 inhibitor LMK-235 at indicated dosages. Quantification results are shown as means ± SEM from three independent experiments (***P < 0.001, by unpaired Student’s t-test). **e** SerRS acetylation in MDA-MB-231 cells treated with indicated dosages of TSA and LMK-235 were analyzed by western blot. Quantification results are shown as means ± SEM from three independent experiments (*P < 0.05, **P < 0.01, by unpaired Student’s t-test). **f** Western blot analysis to show that HDAC4 and HDAC5 in MDA-MB-231 cells were specifically silenced by shRNAs, i.e., shHDAC4 and shHDAC5. A shRNA targeting LacZ (shLacZ) was used as a negative control. Quantification results are shown as means ± SEM from three independent experiments (***P < 0.001, by unpaired Student’s *t*-test). **g﻿** HDAC4 and HDAC5 were silenced by specific shRNAs and the nuclear translocation of SerRS was analyzed by western blot. Quantification results are shown as means ± SEM from three independent experiments (*P < 0.05, by unpaired Student’s *t*-test)

Since the inhibitory effect of SerRS on lipid synthesis depends on its nuclear presence as a transcriptional regulator of key genes involved in lipid synthesis (Fig. 4) and its nuclear translocation is regulated by acetylation on lysine 323 in the presence of glucose (Fig. 1e) which is dysregulated in MDA-MB-231 cells (Fig. 1g), we next investigated the deacetylases involved in the regulation of SerRS acetylation. We first checked the sirtuin family of NAD-dependent protein deacetylase since SerRS has been reported to be able to interact with SIRT2 to repress transcription.^22^ The general sirtuin deacetylase inhibitor nicotinamide did not affect the SerRS nuclear translocation in MDA-MB-231 cells, neither did the specific SIRT1 and SIRT2 inhibitors (Fig. 6a). In MCF 10A cells, inhibition of SIRT1 and SIRT2 also didn’t affect the SerRS nuclear translocation (Fig. 6b). These results ruled out the possibility that SerRS acetylation and nuclear translocation was regulated by sirtuin in breast cancer cells.

We next investigated the classical histone deacetylase (HDAC) family by using specific inhibitors. Interestingly, we observed that the pan-HDAC inhibitor trichostatin A (TSA)^28^ could significantly increase the nuclear import of SerRS (Fig. 6c), suggesting that HDAC family members regulated the SerRS acetylation and nuclear translocation. To further identify which HDAC member is specific for SerRS acetylation, we utilized more specific HDAC inhibitors. As Fig. 6c shown, both HDAC1 and HDAC3-specific inhibitor entinostat^29^ and HDAC6-specific inhibitor tubastatin A^30^ did not affect the SerRS nuclear translocation in MDA-MB-231 cell. However, HDAC4 and HDAC5 (HDAC4/5) specific inhibitor LMK-235^31^ could increase the SerRS nuclear import as TSA did (Fig. 6d), which consistently increased the acetylation of SerRS at lysine 323 (Fig. 6e). And silencing either HDAC4 or HDAC5 was able to promote the nuclear import of SerRS as well (Fig. 6f, g), strongly indicating that HDAC4 and HDAC5 regulate the acetylation of SerRS at lysine 323 and hence its nuclear localization in breast cancer cells.

## Discussion

As essential enzymes catalyzing the first reaction in protein biosynthesis, AARSs expanded their functions beyond protein translation during the evolution by the addition of many non-catalytic domains, which play important roles in a variety of physiological processes including the development of vascular and peripheral neuron system and the regulation of immune responses.^32^ Disruption of these non-catalytic functions of AARSs has been implicated in many disorders, such as neurodegenerative diseases, chronic myeloid leukemia, and solid tumors.^33^ Besides the classical functions of AARSs in protein anabolism, some AARSs have been shown to regulate metabolism and related physiological processes such as angiogenesis that affects the transportation of nutrients. Leucyl-tRNA synthetase can work as a sensor of leucine to mediate the amino acid signaling to the mTORC1, a major regulator of anabolism.^34^ We previously identified that SerRS gained a noncanonical function in the cell nucleus to counteract with c-Myc, an oncogene that regulates angiogenesis and metabolism, for the maintenance of the vascular homeostasis.^22^ However, how the nuclear function of SerRS is regulated and its biological significance remain unknown. Here, we discovered a SerRS mediated pathway that transduces the glucose signal to glucose-fueled lipogenesis, in which high glucose level can increase the acetylation of SerRS at lysine 323, resulting in the translocation of SerRS into the nucleus, where it functions as a transcriptional repressor of many key genes involved in the de novo lipid biosynthesis to inhibit lipogenesis in normal mammary gland epithelial cells. Actually, in most normal cells, except hepatocytes and adipocytes, de novo lipid synthesis is not active and glucose-sourced carbon atoms are not used for lipid synthesis.^35^ The SerRS-mediated pathway reported here may contribute this metabolic feature. Disruption of glucose regulated SerRS acetylation and nuclear translocation was observed in breast cancer cells (Fig. 1c, d and g), in which de novo fatty acid biosynthesis is always reactivated and contributes to many malignant characteristics of cancer cells.^1,2,13^ By using IHC staining, we did observe dramatically decreased SerRS acetylation in human breast cancer specimen compared with normal adjacent cells (Fig. 1o), and overexpression of acetylation-mimetic K323Q mutant SerRS dramatically repressed the proliferation of MDA-MB-231 and the growth of breast cancer xenografts in mice (Fig. 3e–g), supporting that disrupted SerRS acetylation contributes to the progression of breast cancers. Hence, we discovered a mechanism underlying the deregulation of lipid metabolism in breast cancer, which provide potential strategy to restore the lipid homeostasis that has been demonstrated to benefit the therapy of breast cancers.

We also analyzed 9112 breast cancer patients in 17 studies in TCGA database and found that there were eight duplicate mutations of SerRS, i.e., D378Y (variant allele frequency 22%), D244V (42%), I476N (22%), V177G (38%), E458* (*nonsense mutation, 30%), L196F (13%), W159Gfs*17 (FS del, frame shift and ShallowDel, 9%), N54Efs*24 (frame shift with 24-aa insertion, 22%). Among these mutations, I476N mutation is near the nuclear localization signal (NLS) ^481^KKQKKQHEGSKKK^493^ and E458* mutation leads to the deletion of NLS. In our previous work, we have shown that D378 may affect the confirmation of NLS and hence its availability for nuclear translocation.^21^ Taken together, these three mutations may affect the nuclear translocation of SerRS, suggesting the important role of nuclear SerRS in tumorigenesis.

Obesity has been well recognized as a risk factor of cancers growing in adipocyte-rich environments, including gastric, colon and breast cancers.^36^ Obesity-associated adipose tissue microenvironment has been implicated in the alterations in systemic endocrine, inflammation and metabolic interaction between adipocytes and cancer cells that eventually favor the initiation, growth and metastasis of tumors.^37^ Acetylation-defective heterozygote mutation in lysine 323 of SerRS dramatically decreased the nuclear translocation of SerRS in mice (Fig. 1k, l), leading to increased adipose tissue mass (Fig. 2c, e), mimicking the obesity-like phenotype. These data suggest that glucose-regulated nuclear translocation of SerRS also affects the homeostasis of adipose tissue and its malfunction is able to change the normal adipose tissue microenvironment to obesity-like adipose environment, that may also contribute to the initiation and progression of breast cancer. Further studies are needed on Sars^K/R^ mice to evaluate the role of SerRS in shaping the adipose microenvironment and its influence on the initiation and progression of breast cancers.

Although the non-catalytic functions of AARSs have been implicated in many pathologies and are potential therapeutic targets,^33^ it remains challenging to specifically target the non-catalytic activities of AARSs without affecting their classical functions in protein biosynthesis, which may cause side effects when inhibited. In case of SerRS, its anti-tumor function is due to the noncanonical activity in the nucleus as a transcriptional repressor of VEGFA^21,22^ or lipogenic genes (Fig. 4). Understanding the key regulatory mechanism underlying the nuclear translocation of SerRS is of vital importance for the precise application of the anti-tumor activity of SerRS for the therapy of breast cancers. Here, we identified that HDAC4/5 affected the acetylation level of SerRS and hence its nuclear translocation (Fig. 6c–g). HDAC4 and HDAC5 belong to class II HDACs, which exhibit specific roles in the progression of breast cancer.^38^ Elevated expression of HDAC4/5 has been observed in breast cancers, contributing to the progression and drug-resistance of cancer cells.^31,39–42^ The high expression of HDAC4/5 in breast cancer cells might lead to the low acetylation of SerRS that consequently decreases the nuclear translocation of SerRS and inhibits the activity of SerRS in suppressing abnormal lipogenesis. Therefore, the anti-tumor activity of HDAC4/5 inhibitor may be partly caused by increasing SerRS acetylation and nuclear localization. Given that the expression level of SerRS also showed slight decrease in breast cancer (Fig. 1m), the application of HDAC4/5 inhibitors combined with small molecules that increase SerRS expression, such as all-trans retinoic acid and emodin,^43,44^ could achieve promising effects in the therapy of breast cancers.

## Materials and methods

### Chromatin immunoprecipitation and DNA deep sequencing (ChIP-Seq)

ChIP was performed by using a ChIP-IT Express Enzymatic Kit (Active Motif, Carlsbad, CA, USA) according to the manual. Briefly, MDA-MB-231 cells transfected with Flag-tagged SerRS were seeded in 15-cm dishes and grown to 70–80% confluence. Cross-linking was performed with 1% formaldehyde and stopped by incubation with glycine. After the cells were harvested and lysed, the chromatin was sheared with enzymatic shearing cocktail for 10 min at 37 °C and subjected to immunoprecipitation with 2 μg of anti-Flag antibody or mouse IgG (Abmart, Shanghai, China) as a negative control. After washing, immunoprecipitated chromatin was eluted and reverse-crosslinked. DNA was treated with RNase A and proteinase K and purified by phenol/chloroform extraction. High quality purified DNA samples were used to prepare ChIP-Seq libraries according to the NEBNext protocol and sequenced using BGISEQ-500 (Beijing Genomic Institution (BGI), Beijing, China).

### Untargeted lipidomics study

1. **Sample preparation and lipid extraction**
  Cells were seeded in 10-cm dishes and washed twice with cold phosphate-buffered saline (PBS), and then washed with 0.9% sodium chloride solution. Cells were harvested by scrapping with methanol, flash-frozen in liquid nitrogen, and stored at −80 °C. Lipids were extracted according to MTBE method.^45^ Briefly, samples were homogenized with 200 µL water and 240 µL methanol. Then 800 µL of MTBE was added and the mixture was sonicated 20 min at 4 °C, followed by sitting still for 30 min at room temperature. The solution was centrifuged at 14,000 g for 15 min at 10 °C and the upper organic solvent layer was obtained and dried under nitrogen.
2. **LC**–**MS/MS method for lipid analysis**
  Reverse phase chromatography was selected for LC separation using CSH C18 column (1.7 µm, 2.1 mm × 100 mm, Waters). The lipid extracts were re-dissolved in 200 µL 90% isopropanol/acetonitrile, centrifuged at 14,000 g for 15 min, and finally 3 µL of sample was injected. Solvent A was acetonitrile-water (6:4, v/v) with 0.1% formic acid and 0.1 mM ammonium formate and solvent B was acetonitrile-isopropanol (1:9, v/v) with 0.1% formic acid and 0.1 mM ammonium formate. The initial mobile phase was 30% solvent B at a flow rate of 300 μL/min. It was held for 2 min, and then linearly increased to 100% solvent B in 23 min, followed by equilibrating at 5% solvent B for 10 min.
  Mass spectra (MS) was acquired by Q-Exactive Plus in positive and negative mode, respectively. ESI parameters were optimized and preset for all measurements as follows: Source temperature, 300 °C; Capillary Temp, 350 °C. In positive ion mode, the ion spray voltage was set at 3000 V, S-Lens RF Level was set at 50% and the scan range of the instruments was set at m/z 200–1800. In negative ion mode, the ion spray voltage was set at −2500 V, S-Lens RF Level was set at 60% and the scan range of the instruments was set at m/z 250–1800.
3. **Identification by lipid search**
  “Lipid Search” is a search engine for the identification of lipid species based on MS/MS math. Lipid Search contains more than 30 lipid classes and more than 1,500,000 fragment ions in the database. Both mass tolerance for precursor and fragment were set to 5 ppm. The displayed product ion threshold was selected as 5 and grades A, B, C, D were all used for ID quality filter.

### Metabolic flux assay

Cells were cultured in glucose-free and glutamine-free DMEM (Thermo-Fisher Scientific, Waltham, MA, USA) containing 5% fetal bovine serum (Biological Industries, Israel), 100 U/mL penicillin and 100 μg/mL (Thermo-Fisher Scientific), and supplemented with glucose and the tracer [U-^13^C_6_] glucose (Cambridge Isotope Labs, Tewksbury, MA, USA) for 5 days.

The cells were then rinsed three times with PBS and collected in tubes by scraping with 1 mL PBS. 4 mL of CHCl_3_:MeOH (methanol) (2:1) mixture was added and vortexed three times, then centrifuged at 3000 rpm for 15 min. The organic phase was transferred to a glass vial and dried. The samples were then quantified with BCA Protein Assay Kit (ThermoFisher Scientific).

For fatty acid analysis, samples were re-suspended in 100 uL of dichloromethane CH_2_Cl_2_:MeOH (v: v = 1:1). The UPLC system was coupled to a Q-Exactive orbitrap mass spectrometer (Thermo Fisher, CA) equipped with a heated electrospray ionization (HESI) probe. Lipid extracts were separated by a CORTECS C18 (100 × 2.1 mm 1.6 μm) column (Waters, USA). A binary solvent system was used, in which mobile phase A consisted of ACN:H_2_O (60:40) and 10 mM Ammonium acetate, and mobile phase B of IPA:ACN (90:10). An 18-minute gradient with flow rate of 220 μL/min was used. Linear gradient was as follows: 0 min, 30% B; 2.5 min, 30% B; 8 min, 50% B; 10 min, 98% B; 15 min, 98% B; 15.1 min, 30% B; 18 min, 30% B. Column chamber and sample tray were held at 40 °C and 10 °C, respectively. Data with mass ranges of m/z 150-2000 was acquired at negative ion mode. The full scan was collected with resolution of 70,000. The source parameters are as follows: spray voltage: 3000 V; capillary temperature: 320 °C; heater temperature: 300 °C; sheath gas flow rate: 35 Arb; auxiliary gas flow rate: 10 Arb. Data analysis and lipid identification were performed by the TraceFinder (Thermo-Fisher Scientific) according to endogenous MS database by accurate masses.

### Statistical analysis

All statistical analysis was conducted using Prism 5.0 software (GraphPad Software, San Diego, CA, USA) and the most statistical analysis were using two-tailed Student’s t-tests, except that the statistical analysis on cell proliferation and xenograft growth was using two-way ANOVA.

## Supplementary information

Supplementary material

Supplementary Table S1

Supplementary Table S2

Supplementary Table S3

Supplementary Table S4

## Data Availability

All the datasets presented in the paper are available from the corresponding author upon reasonable request.
