# Supplementary material for: Glucose-sensitive acetylation of Seryl tRNA synthetase regulates lipid synthesis in breast cancer
Source: Signal Transduct Target Ther. 2021 Aug 16;6:303. doi: 10.1038/s41392-021-00714-0 (PMC8368063; doi:10.1038/s41392-021-00714-0)
Supplement: Supplementary file 1 — Supplementary material [file 41392_2021_714_MOESM1_ESM.pdf]

Supplementary Materials for

Glucose-sensitive acetylation of Seryl tRNA synthetase regulates lipid synthesis in breast cancer

Jin Zhao[1], Hangming Bai[1], Xiaoyu Li[1], Jie Yan[1], Gengyi Zou[1], Longlong Wang[1], Xiru Li[3], Ze Liu[2], Rong Xiang[1], Xianglei Yang[2] and Yi Shi[1*]

[1]School of Medicine, Nankai University, Tianjin 300071, China

[2]The Scripps Research Institute, La Jolla, CA 92037, USA

[3]Department of General Surgery, Chinese PLA General Hospital, Beijing 100853, China

Correspondence to: Yi Shi (yishi@nankai.edu.cn)

This PDF file includes:

Materials and Methods
Figures. S1 to S3

Other Supplementary Materials for this manuscript include the following:

Tables S1 to S4
Supplementary Table S1 (Excel document)
Supplementary Table S2 (Excel document)
Supplementary Table S3 (Excel document)
Supplementary Table S4 (Excel document)

Materials and methods

Cell culture and constructs

All cells were cultured in a humidified incubator at 37 ℃ with 5% CO_2. MDA-MB-231 cells (purchased from American Type Culture Collection, ATCC) were cultured in Dulbecco's modified Eagle's medium (DMEM) with 4.5 g/L glucose (Biological Industries, Israel), supplemented with 10% fetal bovine serum (FBS) (Biological Industries, Israel), 100 U/mL penicillin, and 100 μg/mL streptomycin (Thermo-Fisher Scientific, Waltham, MA, USA). MCF 10A cells (from ATCC) were maintained in DMEM/F12 supplemented with 5% horse serum, 100 U/mL penicillin, 100 μg/mL streptomycin, 20 ng/mL EGF, 0.5 μg/mL hydrocortisone, and 100 ng/mL cholera toxin. Stably transfected MDA-MB-231 cells were made by using lentivirus, which was prepared by using the pLV-EF1α-IRES-bsd plasmid (Biosettia, San Diego, CA, USA), followed by selection using blasticidin to generate stable polyclonal cell line with SerRS overexpression and the control. Mutant SerRS constructs were generated by site-directed mutagenesis. The Tet-inducible SerRS expression cells were established by using the ViraPower HiPerform T-rex Gateway Vector Kit (Thermo-Fisher Scientific, Waltham, MA, USA) according to the manufacturer's instructions. Briefly, MDA-MB-231 cells were infected with pLenti3.3/TR virus expressing the tetracycline repressor gene and selected with G418 (500 μg/mL; Sangon Biotech, Shanghai, China). The cells were infected with pLenti6.3/ TO/V5-DEST virus expressing SerRS and selected with 5 μg/mL blasticidin (Thermo-Fisher Scientific, Waltham, MA, USA). SerRS expression was induced by treating the cells with 0.5 μg/mL doxycycline (Sigma, St. Louis, MO, USA).

Animal studies

$Sars^{K/R}$ knock-in mice in the C57BL/6J background were generated by Beijing Biocytogen (Beijing, China). And all mice were maintained on a 12-hour-light /12-hour-dark cycle in a pathogen-free barrier facility with free access to water and regular chow diet. All mice experiments were performed according to the guidelines established by the Nankai University Animal Care and Use Committee (NUACUC) by skilled experimenters under an approved protocol, which was in accordance with the principles and procedures outlined in the NIH Guide for the Care and Use of Laboratory Animals.

Breast cancer xenograft mouse models

Six-week-old female NOD/SCID mice or BALB/c mice (Beijing HFK Bioscience, Beijing, China) were subcutaneously injected under the second right mammary fat pad of each mouse with $2×10^6$ of MDA-MB-231 cells or $1×10^5$ of 4T1 cells respectively. The tumor size was measured every 2-3 days. Tumor volumes were estimated using the formula: volume = $(\text{width})^2 \times (\text{length})/2$, where the width is the smaller of the two dimensions. All mouse experiments were performed according to the guidelines established by the Nankai University Animal Care and Use Committee (NUACUC).

Cell fractionation analysis

Cell fractionation was performed according to the previously described protocol [1]. Briefly the cells were harvested with 0.25% Trypsin-EDTA, and the cytoplasmic fraction was separated and extracted with swelling buffer (10 mM Tris-HCl pH 7.4, 2 mM EDTA, proteinase inhibitor cocktail (Roche, Basel, Switzerland)) and followed by the addition of 1/10 volume of plasma membrane lysis buffer (10 mM Tris-HCl pH 7.1, 2 mM $MgCl_2$, 1% Triton X-100), the nuclear fractions were then extracted by using nuclear extraction buffer (20 mM HEPES pH 7.6, 300 mM NaCl, 2 mM EDTA, 1 mM 1,4-Dithiothreitol, 10% glycerol, 1% Triton X-100, protease inhibitor cocktail). SerRS was detected with an anti-SerRS antibody (self-made in rabbit using recombinant human SerRS protein). An anti-Lamin A/C antibody (Proteintech, Wuhan, China) and an α-tubulin antibody (Proteintech, Wuhan, China) were used as nuclear and cytoplasmic markers, respectively, to test the purities.

Western blot

The cells were washed with phosphate-buffered saline (PBS) and lysed with cell lysis buffer (20 mM Tris-HCl pH 7.5, 150 mM NaCl, 1 mM of EDTA, 1 mM EGTA, 1% Triton X-100, 2.5 mM sodium pyrophosphate, 1 mM beta-glycerophosphate, 1 mM Na_3VO_4, and protease inhibitor cocktail) supplemented with protease inhibitor cocktail. Protein concentration of each sample was quantified with BCA Protein Assay Kit (ThermoFisher Scientific). Equal amounts of protein extracts were mixed with SDS loading buffer and boiled at 95℃ for 5 minutes. Samples were loaded on 12% Tris-Acrylamide gels, then transferred onto polyvinylidene difluoride membranes using the Trans-Blot SD SEMI-DRY TRANSFER CELL (Bio-Rad, Hercules, CA, USA). Membranes were then blotted with indicated antibodies and secondary horseradish peroxidase-conjugated antibodies (ZSGB-BIO, Beijing, China; at the dilution of 1:5000). Blots were visualized with ECL chemiluminescence kit (Millipore, Billerica, MA, USA).

RNA extraction, quantitative RT–PCR, and deep RNA sequencing

Total RNA was extracted directly from cultured cells using the TRIzol reagent (ThermoFisher Scientific), and then reverse-transcribed into cDNAs with M-MLV reverse transcriptase (Promega, Madison, WI, USA) and oligo dT(20) as the primer. mRNA levels were then quantified by quantitative PCR using the Hieff® qPCR SYBR Green Master Mix (Yeasen, Shanghai, China) on a QuantStudio3 (ThermoFisher Scientific). Relative expression levels were calculated after being normalized to β-actin (ACTB). The sequences of the primer were listed in the following table:

Genes	Sequences of Forwar primers (5' to 3')	Sequences of reverse primers (5' to 3')
ACLY	CGGCAGAGGTAGAGCAGGTC	GGTTGACCCCAACGAGACCA
ACACA	AGCCGCTTGCCTGACTTTTG	TGCAACAGGGGTGGAGATGG
ACACB	AAACCTCATCCCGAGCCAGG	TGTGGCCTCCAGACCTTGTG
ACSS1	GGCAGACAGAAACAGGTGGC	AATCGCTGGTGGTCGCCATA
ACSS2	GACGTGATGGGGCTTCCTGA	TACCGAAGGAATGGGCCAGG
HMGCS1	CGGACTGTCCTTTCGTGGCT	GGCCAGCAAGCTTCTGCATT
HMGCR	CCTTCCGCTCCGCGACT	AGGGATGGGAGGCCACAAAG
ACAT2	AGATCCTGTGGTCATCGTCTCG	CTGCACCCACACTGGCTTGT
ACTB	CGTCACCAACTGGGACGA	ATGGGGGAGGGCATACC

For deep RNA sequencing, the quality of the RNA was determined using an Agilent 2100 Bioanalyzer, and samples with RNA integrity numbers (RINs) over 8 were used to construct the libraries and sequenced on BGISEQ-500 platforms (BGI, Wuhan, China).

Immunofluorescence staining

Cells were seeded on coverslips at the density of 5 x10^4 cells. After 24 hours, cells were fixed in 4% paraformaldehyde (Sigma-Aldrich, St. Louis, MO, USA) for 15 minutes at room temperature. The cells were then permeabilized with 0.15% Triton X-100 for 15 min at room temperature, followed by incubation in the blocking buffer (5% goat serum, 0.3% Triton X-100, PBS) for 1 h. Cells were incubated at 4 °C overnight with anti-SerRS (self-made and affinity-purified; at 1:200 dilution) or anti-Ac-SerRS (customized in Proteintech, Wuhan, China; at a dilution of 1:200 dilution) antibodies. After three times of washes with PBS, fluorescent secondary antibody Alexa Fluor goat anti-rabbit 488 at a dilution of 1:200 was applied to cells and incubated at room temperature in dark for 1 hour. Cells were then washed three times with PBS and counterstained with 0.1 μg/mL 4,6-diamidino-2-phenylindole (DAPI, Sigma-Aldrich), phalloidine (Sangon Biotech, China; at 1:200 dilution) or Nile red (Yeasen, Shanghai, China; 1 μg/mL) and mounted on glass slides with ProlongGold Antifade Reagent (Thermo-Fisher). The cells were visualized using a confocal microscope (Olympus, Tokyo, Japan).

Immunohistological staining

Formalin-fixed, paraffin-embedded tissue sections were prepared. After deparaffinizing with xylene and rehydrating through graded ethanol, antigen retrieval was conducted for 1 hour using a steam pot in 10 mM citrate buffer (pH 6.0), followed by HRP inactivation in 3% hydrogen peroxide for 10 min and blocking in PBST buffer (PBS, 0.3% Triton X-100) containing 5% goat serum. The tissue sections were then blotted with anti-SerRS antibody (at 1:500 dilution) in PBST buffer or with anti-Ac-SerRS antibody (at 1:200 dilution) in Tris-EDTA buffer (10 mM Tris base, 1 mM EDTA solution, 0.05%

Tween 20, pH 9.0) at 4°C overnight. After incubation with the secondary antibodies, the tissue slides were visualized by staining with 3,3'-Diaminobenzidine DAB kit (ZSGB-BIO, Beijing, China). The images were recorded by an Olympus BX51 Epi-fluorescent microscope (Olympus, Tokyo, Japan). The H-score was used for the quantification of each protein, which was calculated by the multiplication of percentage of cells that stained positive (0% – 100%) and staining intensity on a numerical scale (none=0, weak=1, moderate=2, strong=3).

References

1 Shi, Y., Wei, N. & Yang, X. L. Studying nuclear functions of aminoacyl tRNA synthetases. *Methods* **113**, 105-110, doi:10.1016/j.ymeth.2016.09.011 (2017).

Supplementary figures

Figure S1

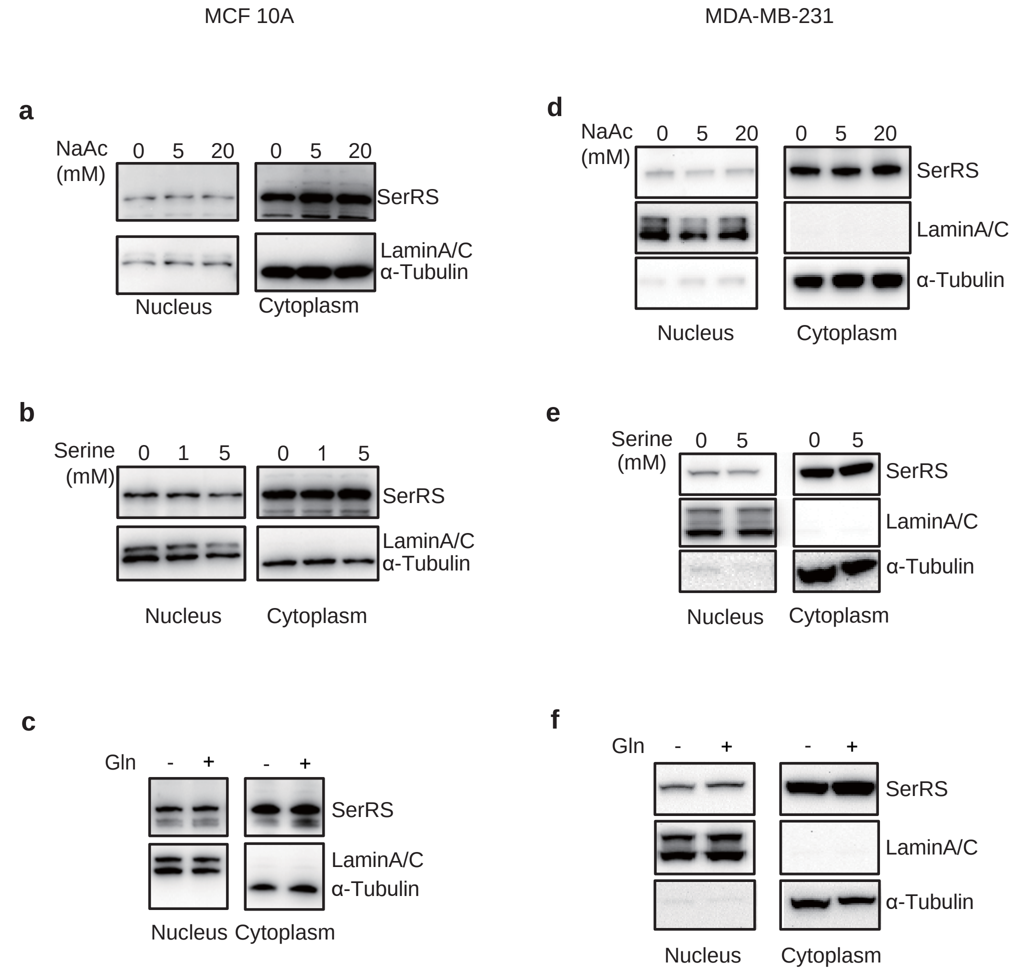

Figure S1. The nuclear translocation of SerRS in cells fed with different nutrients.
(a-c) Western blot analysis of SerRS in the nuclear and cytoplasm fractions of MCF 10A cells cultured in indicated dosages of sodium acetate (NaAc) (a), serine (b) and glutamine (Gln) (c) for 48 hours.
(d-f) Western blot analysis of SerRS in the nuclear and cytoplasm fractions of MDA-MB-231 cells cultured in indicated dosages of sodium acetate (NaAc) (d), serine (e) and glutamine (Gln, 2 mM) (f) for 48 hours.

Figure S2

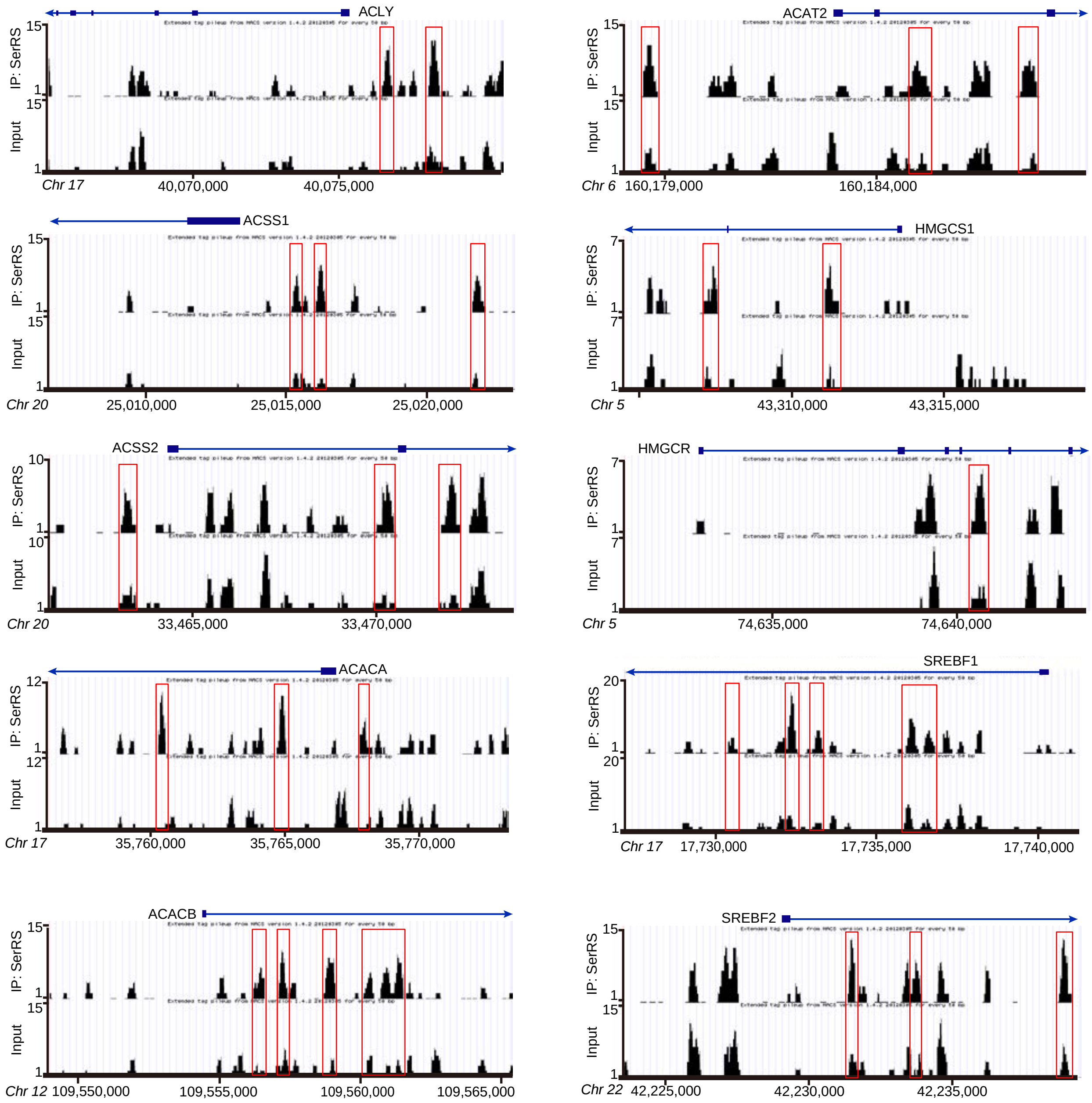

Figure S2. SerRS binding sites on key genes involved in the de novo lipid synthesis. MDA-MB-231 cells were transfected with Flag-tagged SerRS and the SerRS-bound DNA fragments were immunnoprecipitated by anti-Flag antibody and analyzed deep sequencing. SerRS binding sites were identified on the indicated key genes involved in the de novo lipid synthesis.

Figure S3

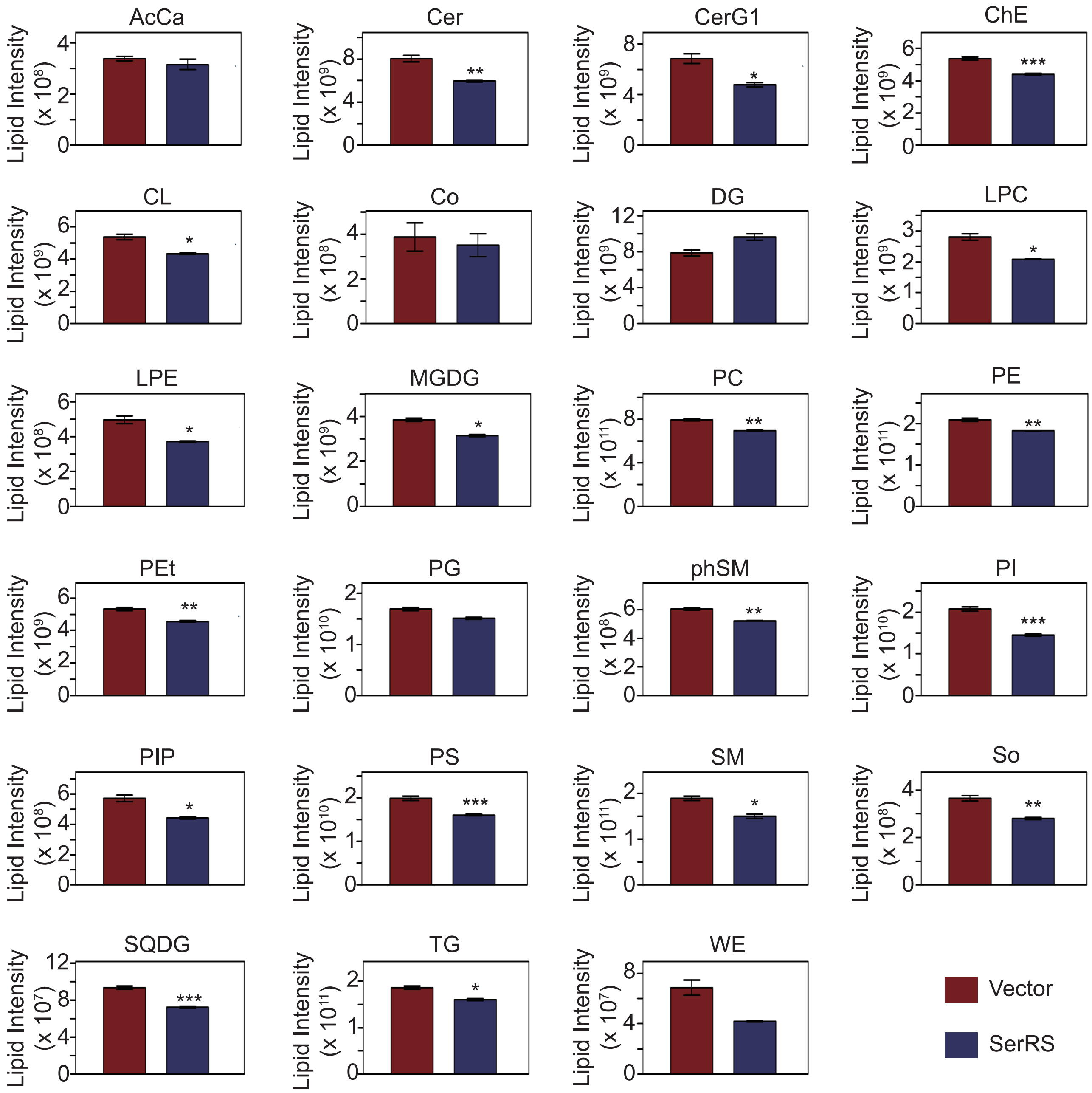

Figure S3. SerRS systematically inhibits the lipid synthesis in breast cancer cells.
All identified lipid species belonging to indicated lipid classes were quantified by untargeted lipidomics analysis on SerRS-overexpressed MDA-MB-231 cells or control cells transfected with empty vector. Data are shown as means ± SEM, n=6, *P<0.05, **P<0.01, ***P<0.001, by tow-tailed Student's t-test.
