# Supplementary material for: Glucose-sensitive acetylation of Seryl tRNA synthetase regulates lipid synthesis in breast cancer
Source: Signal Transduct Target Ther. 2021 Aug 16;6:303. doi: 10.1038/s41392-021-00714-0 (PMC8368063; doi:10.1038/s41392-021-00714-0)
Supplement: Supplementary file 5 — Supplementary Table S4 [file 41392_2021_714_MOESM5_ESM.pdf]

Expected RT	m/z (Expected)	Compound	Con-	Tet-OFF	Tet-ON
1.82	199.1698	FA(12:0)*C12H24O2	0.00E+00	1.74E+07	1.26E+07
1.82	200.1732	FA(12:0)*m1	0.00E+00	1.70E+06	2.50E+05
2.84	227.2011	FA(14:0)*C14H28O2	0.00E+00	7.52E+07	3.93E+07
2.84	228.2045	FA(14:0)*m1	0.00E+00	6.42E+05	0.00E+00
2.09	221.1542	FA(14:3)*C14H22O2	0.00E+00	1.01E+08	5.26E+07
2.09	222.1575	FA(14:3)*m1	0.00E+00	9.70E+05	2.97E+06
3.7	241.2168	FA(15:0)*C15H30O2	0.00E+00	2.79E+07	1.22E+07
3.7	242.2201	FA(15:0)*m1	0.00E+00	6.12E+05	3.77E+05
4.85	255.2324	FA(16:0)*C16H32O2	0.00E+00	3.14E+09	1.88E+09
4.85	256.2358	FA(16:0)*m1	0.00E+00	1.10E+07	2.54E+06
4.85	257.2391	FA(16:0)*m2	0.00E+00	4.23E+06	1.50E+06
4.85	258.2425	FA(16:0)*m3	0.00E+00	3.23E+06	1.94E+06
4.85	259.2458	FA(16:0)*m4	0.00E+00	5.04E+06	2.16E+06
4.85	260.2492	FA(16:0)*m5	0.00E+00	1.73E+06	6.73E+05
4.85	261.2525	FA(16:0)*m6	0.00E+00	1.16E+07	5.35E+06
4.85	262.2559	FA(16:0)*m7	0.00E+00	2.70E+06	1.83E+06
4.85	263.2592	FA(16:0)*m8	0.00E+00	1.64E+07	7.62E+06
4.85	264.2626	FA(16:0)*m9	0.00E+00	4.64E+06	1.89E+06
4.85	265.2659	FA(16:0)*m10	0.00E+00	2.01E+07	8.61E+06
4.85	266.2693	FA(16:0)*m11	0.00E+00	3.49E+06	1.30E+06
4.85	267.2726	FA(16:0)*m12	0.00E+00	1.42E+07	5.77E+06
4.85	268.276	FA(16:0)*m13	0.00E+00	1.78E+06	9.08E+05
4.85	269.2793	FA(16:0)*m14	0.00E+00	9.35E+06	3.91E+06
3.31	253.2168	FA(16:1)*C16H30O2	0.00E+00	2.45E+07	7.56E+06
3.31	254.2201	FA(16:1)*m1	0.00E+00	0.00E+00	0.00E+00
6.12	269.2481	FA(17:0)*C17H34O2	0.00E+00	3.83E+07	1.75E+07
6.12	270.2514	FA(17:0)*m1	0.00E+00	0.00E+00	1.03E+06
7.43	283.2637	FA(18:0)*C18H36O2	0.00E+00	3.22E+09	1.88E+09
7.43	284.2671	FA(18:0)*m1	0.00E+00	1.67E+06	4.05E+06
7.43	285.2704	FA(18:0)*m2	0.00E+00	3.21E+07	1.17E+07
7.43	286.2738	FA(18:0)*m3	0.00E+00	8.09E+06	4.55E+06
7.43	287.2771	FA(18:0)*m4	0.00E+00	4.37E+06	1.77E+06
7.43	288.2805	FA(18:0)*m5	0.00E+00	9.79E+05	4.44E+05
7.43	289.2838	FA(18:0)*m6	0.00E+00	6.29E+06	2.72E+06
7.43	290.2872	FA(18:0)*m7	0.00E+00	2.13E+06	9.30E+05
7.43	291.2905	FA(18:0)*m8	0.00E+00	1.37E+07	6.81E+06
7.43	292.2939	FA(18:0)*m9	0.00E+00	3.67E+06	1.42E+06
7.43	293.2972	FA(18:0)*m10	0.00E+00	2.15E+07	8.53E+06
7.43	294.3006	FA(18:0)*m11	0.00E+00	8.95E+06	3.00E+06
7.43	295.3039	FA(18:0)*m12	0.00E+00	1.84E+07	8.09E+06
7.43	296.3073	FA(18:0)*m13	0.00E+00	4.40E+06	2.04E+06

7.43	297.3106	FA(18:0)*m14	0.00E+00	1.43E+07	6.31E+06
7.43	298.314	FA(18:0)*m15	0.00E+00	2.79E+06	7.86E+05
7.43	299.3173	FA(18:0)*m16	0.00E+00	5.53E+06	2.67E+06
5.3	281.2481	FA(18:1)*C18H34O2	0.00E+00	3.15E+08	1.50E+08
5.3	282.2514	FA(18:1)*m1	0.00E+00	2.46E+05	0.00E+00
5.3	287.2682	FA(18:1)*m6	0.00E+00	1.96E+06	7.18E+05
5.3	289.2749	FA(18:1)*m8	0.00E+00	3.28E+06	1.17E+06
5.3	290.2782	FA(18:1)*m9	0.00E+00	9.30E+05	0.00E+00
5.3	291.2816	FA(18:1)*m10	0.00E+00	8.51E+06	3.39E+06
5.3	292.2849	FA(18:1)*m11	0.00E+00	1.10E+06	3.00E+05
5.3	293.2883	FA(18:1)*m12	0.00E+00	7.57E+06	0.00E+00
3.62	279.2324	FA(18:2)*C18H32O2	0.00E+00	1.07E+08	5.27E+07
3.62	280.2358	FA(18:2)*m1	0.00E+00	0.00E+00	1.01E+06
8.74	297.2794	FA(19:0)*C19H38O2	0.00E+00	1.22E+07	5.16E+06
8.74	298.2827	FA(19:0)*m1	0.00E+00	0.00E+00	1.04E+06
9.86	311.295	FA(20:0)*C20H40O2	0.00E+00	1.31E+08	7.00E+07
9.86	312.2984	FA(20:0)*m1	0.00E+00	0.00E+00	3.12E+06
9.86	313.3017	FA(20:0)*m2	0.00E+00	1.17E+07	4.47E+06
7.7	309.2794	FA(20:1)*C20H38O2	0.00E+00	2.77E+07	1.33E+07
7.7	310.2827	FA(20:1)*m1	0.00E+00	2.58E+06	3.05E+06
3.31	303.2324	FA(20:4)*C20H32O2	0.00E+00	7.69E+06	2.62E+06
3.31	304.2358	FA(20:4)*m1	0.00E+00	3.49E+05	8.45E+05
3.31	305.2391	FA(20:4)*m2	0.00E+00	3.59E+05	0.00E+00
10.65	339.3263	FA(22:0)*C22H44O2	0.00E+00	2.11E+08	1.69E+08
10.65	340.3297	FA(22:0)*m1	0.00E+00	3.39E+06	2.55E+06
10.65	341.333	FA(22:0)*m2	0.00E+00	5.27E+06	4.90E+06
9.88	337.3107	FA(22:1)*C22H42O2	0.00E+00	1.20E+07	6.01E+06
9.88	338.314	FA(22:1)*m1	0.00E+00	1.52E+06	1.28E+06
3.64	329.2481	FA(22:5)*C22H34O2	0.00E+00	8.90E+06	3.49E+06
3.64	330.2514	FA(22:5)*m1	0.00E+00	3.01E+05	2.75E+05
2.79	327.2324	FA(22:6)*C22H32O2	0.00E+00	5.68E+06	1.61E+06
2.79	328.2358	FA(22:6)*m1	0.00E+00	2.06E+05	0.00E+00
10.81	353.342	FA(23:0)*C23H46O2	0.00E+00	9.02E+07	7.06E+07
10.81	354.3453	FA(23:0)*m1	0.00E+00	0.00E+00	0.00E+00
10.95	367.3576	FA(24:0)*C24H48O2	0.00E+00	2.78E+08	2.35E+08
10.95	368.361	FA(24:0)*m1	0.00E+00	3.07E+06	0.00E+00
10.95	369.3643	FA(24:0)*m2	0.00E+00	1.29E+07	9.61E+06

10.95	371.371	FA(24:0)*m4	0.00E+00	1.50E+07	1.20E+07
10.95	372.3744	FA(24:0)*m5	0.00E+00	4.19E+06	3.33E+06
10.95	373.3777	FA(24:0)*m6	0.00E+00	1.06E+07	1.02E+07
10.63	365.342	FA(24:1)*C24H46O2	0.00E+00	5.21E+07	2.85E+07
10.63	366.3453	FA(24:1)*m1	0.00E+00	2.70E+06	2.23E+06
11.05	381.3733	FA(25:0)*C25H50O2	0.00E+00	1.02E+08	6.98E+07
11.05	382.3766	FA(25:0)*m1	0.00E+00	0.00E+00	1.88E+05
11.16	395.3889	FA(26:0)*C26H52O2	0.00E+00	1.53E+08	9.14E+07
11.16	396.3923	FA(26:0)*m1	0.00E+00	2.61E+06	0.00E+00
11.16	397.3956	FA(26:0)*m2	0.00E+00	1.65E+07	1.15E+07
11.16	398.399	FA(26:0)*m3	0.00E+00	4.40E+06	3.29E+06
11.16	399.4023	FA(26:0)*m4	0.00E+00	1.70E+07	1.34E+07
11.16	400.4057	FA(26:0)*m5	0.00E+00	5.19E+06	3.66E+06
11.16	401.409	FA(26:0)*m6	0.00E+00	1.65E+07	1.31E+07
11.16	402.4124	FA(26:0)*m7	0.00E+00	4.52E+06	3.37E+06
11.16	403.4157	FA(26:0)*m8	0.00E+00	1.12E+07	7.67E+06
11.16	404.4191	FA(26:0)*m9	0.00E+00	2.04E+06	1.61E+06
11.16	405.4224	FA(26:0)*m10	0.00E+00	6.89E+06	4.12E+06
11.16	411.4425	FA(26:0)*m16	0.00E+00	9.25E+06	6.66E+06
11.16	412.4459	FA(26:0)*m17	0.00E+00	2.07E+06	1.26E+06
10.93	393.3733	FA(26:1)*C26H50O2	0.00E+00	2.23E+07	1.93E+07
10.93	396.3833	FA(26:1)*m3	0.00E+00	2.71E+06	2.45E+06
10.93	397.3867	FA(26:1)*m4	0.00E+00	8.33E+06	7.14E+06
10.93	398.39	FA(26:1)*m5	0.00E+00	2.01E+06	1.69E+06
10.93	399.3934	FA(26:1)*m6	0.00E+00	7.03E+06	6.66E+06
10.93	400.3967	FA(26:1)*m7	0.00E+00	1.64E+06	9.78E+05
10.93	401.4001	FA(26:1)*m8	0.00E+00	5.98E+06	2.19E+06
11.31	423.4202	FA(28:0)*C28H56O2	0.00E+00	6.33E+07	4.95E+07
11.31	424.4236	FA(28:0)*m1	0.00E+00	0.00E+00	0.00E+00
11.42	451.4515	FA(30:0)*C30H60O2	0.00E+00	3.21E+07	1.75E+07
11.42	452.4549	FA(30:0)*m1	0.00E+00	0.00E+00	0.00E+00
